# Geographical Distribution of Dental Caries

**Published:** 1886-01

**Authors:** E. G. Betty

**Affiliations:** Cincinnati


### Geographical Distribution of Dental Caries.

E. G. BETTY D.D.S., CINCINNATI.

I have heretofore hesitated to bring before this body the subject now up for consideration, for the reason that I was doubtful of the practicability of carrying out such a plan as I propose. I have since learned that Dakota and Illinois have taken some action in this regard, and that has encouraged me to present at this the first meeting of the Reorganized State Dental Society of Ohio, a few suggestions as to our duty in the premises, and the manner of accomplishing it.

As all are well aware, data collected from divers sources, tabulated and systematised, becomes what is called statistics, and statistics now-a-days, comprise the bulk of our verified and available knowledge. For this reason it becomes necessary for us to collect all the information we can upon the subject of the geographical distribution of dental caries in our State, in order that we may gather from its studious consideration such knowledge as will enable us to bring to bear upon the disease we assume to treat, a more discriminating intelligence of its etiology progress and results.

This we can only do when we have at our command a mass of comprehensive details that will enable us to account for phases, conditions and symptoms that give no surface indication of their origin, yet, when all the antecedents are scrutinized, much that is obscure reveals itself to our gaze, and in the nature of things, becomes more readily amenable to treatment.

Our views are daily becoming broader and deeper, and we seek information from many sources that heretofore have had but little weight in our councils. Dental Caries is not so simple a thing as it would seem to be; it is not only a question of the decomposition of solids and fluids liberating gasses that combine to form acids that break down, dissolve or disintegrate tooth-substance; neither is it solely a matter of the formation of an acid by fermentive fungi. It may be one or other of these, or both, or neither. It is because we don't know this that we must look further into the antecedents of the individual, his family, his race, his habitat, his food, the climate he lives in, the annual rainfall, the maximum, minimum and mean yearly temperature, humidity, etc. These are the things we must take into consideration, and we can do it if we will, and without such a large measure of work for any one of us as to discourage us by its amount or deter us from engaging in it.

Concerted effort will accomplish almost anything, and in this matter, will add a stone to the foundation of the profession we are building. Let us build well while we are at it.

With these few remarks as an introduction, I now wish to call your attention to a blank I have prepared for recording in a tabulated form, the condition of each mouth as regards decay of the teeth. I think I have succeeded in getting a form that is at once simple, complete and exact. The first column to the left is for the number of the examination or case, whichever you choose to call it; the second column for the age of the patient, the third, for sex, the fourth is for the nationality or parentage, as in this country there are vast numbers who are native born, yet possess none of the characteristics of the true American.

All the columns numbered from 1 to 32 inclusive, are for the complete denture of adult life, each tooth thus having a separate space and a distinct number. No. 1 represents the third molar on the upper right side, starting from which point the succeeding numbers represent all the teeth of the upper jaw, in their order around to the third molar on the left side, designated as No. 16;

### EXAMINATION FOR DENTAL CARIES.

C—Crown.
Ap—Anterior proximal.
Pp—Posterior proximal.
La—Labial.
B—Buccal.
P—Palatal.
Li—Lingual.
X—Missing teeth.

} Use these letters to designate the location of the cavity.

*Conducted by Dr.*________________

*Town*________________

*County*________________

*State*________________

*Date*________________

| | AGE. | SEX. | NATIONALITY. | 1 | 2 | 3 | 4 | 5 | 6 | 7 | 8 | 9 | 10 | 11 | 12 | 13 | 14 | 15 | 16 | 17 | 18 | 19 | 20 | 21 | 22 | 23 | 24 | 25 | 26 | 27 | 28 | 29 | 30 | 31 | 32 | REMARKS. |
|---|---|---|---|---|---|---|---|---|---|---|---|---|---|---|---|---|---|---|---|---|---|---|---|---|---|---|---|---|---|---|---|---|---|---|---|---|
| 1 | | | | | | | | | | | | | | | | | | | | | | | | | | | | | | | | | | | | |
| | | | | | | | | | | | | | | | | | | | | | | | | | | | | | | | | | | | | |
| 3 | | | | | | | | | | | | | | | | | | | | | | | | | | | | | | | | | | | | |
| 4 | | | | | | | | | | | | | | | | | | | | | | | | | | | | | | | | | | | | |
| 5 | | | | | | | | | | | | | | | | | | | | | | | | | | | | | | | | | | | | |
| 6 | | | | | | | | | | | | | | | | | | | | | | | | | | | | | | | | | | | | |
| 7 | | | | | | | | | | | | | | | | | | | | | | | | | | | | | | | | | | | | |

returning, the teeth of the lower jaw are numbered from 17, corresponding to the third molar of the left side, around to No. 32, the third molar of the right side. According to this nomenclature, numbers 1, 2, and 3 represent the molars of the upper right side; numbers 4 and 5, the bicuspids, number 6 the cuspid, and 7 and 8 the lateral and central incisors, all of the right side and so on for the thirty-two teeth in the order mentioned.

The last column denominated "remarks," is for the purpose of noting any peculiarity of the individual, his health, temperament, quality of teeth, etc.

To locate the decay very accurately, and yet in a simple manner without consuming an unnecessary amount of time. The various surfaces of the teeth are designated by the first letter, as:

C.—Crown.
Ap.—Anterior Proximal.
Pp.—Posterior Proximal.
La.—Labial.
B.—Buccal.
P.—Palatal.
Li.—Lingual.
X.—Missing teeth.

The cross is used to point out such teeth as are missing, and is written in the column corresponding to the teeth that are absent in the particular case.

There is space on the blank provided for the dentist making the record of the case, to put his name, town, county and State, and the date of examination approximately, month and year being sufficient.

One line across the blank, from left to right, is all that is needed to make a full record of each case, and with the least possible expenditure of time. A probable objection with many, is the time consumed in making such a record of cases, but a little familiarity with the figures corresponding to the teeth, will greatly facilitate the examination and reduce to a minimum the amount of time required to record it upon the blank.

It often happens that a dentist will consume a large part of a day in merely examining the mouths of people who present themselves at his office, a good many of whom do not return, neither do they remunerate the dentist for the time he has spent upon them. If, however, they see him make a record of the examination of their mouths, he will not infrequently be asked if there is any charge, and in that way he may pick up many a stray dollar, and besides have the satisfaction of assisting his State Society in an important work, in addition to having a complete record of the amount of work to be done in any given mouth, in case he should be called upon to make an estimate as to the probable cost. This may be an inducement to many a dentist to fill these blanks where otherwise he would not care to undertake it, preferring to leave it to his more ambitious neighbor, who regularly attends the Society meetings.

It is unnecessary for me at this time, to enumerate the many uses to which such a body of statistics can be put, or to mention the manner in which they are to be studied, further than that they are to serve a scientific purpose, when studied in connection with locality, climate, food, etc.

If the Society sees fit to consider this matter, and will take it in hand, its large membership and larger influence will enable it in the short space of a couple of years to collect statistics from several hundred thousand cases.

The blanks can be prepared at a small outlay, and distributed either by the Corresponding Secretary or some one appointed for the purpose. He can also receive or collect them from the various members after having been filled out and return them to the Society. The labor of compiling the totals and arranging reports by counties, will be somewhat large, and ought to be divided amongst a committee appointed or elected for the purpose. However, these matters will largely determine themselves as they arise.

This collecting of statistics is very necessary, and is being taken in hand by one or two other States, so that it is important for Ohio to follow as soon as possible. It will not be a great while before other States will fall into line, and then the profession will be in possession of a mass of information of incalculable value.

